# Yellow Fever Germs

**Published:** 1888-11

**Authors:** 


### YELLOW FEVER GERMS.

Major G. M. Sternberg, Surgeon U. S. A., went to Havana in May last, in compliance with instructions from the President, to continue the investigation begun last year in Brazil and in Mexico. Through the courtesy of the Spanish army surgeons at the military hospital in Havana he was able to obtain as many autopsies as he required, and made a careful search of the blood and the various organs of the body with reference to the presence of germs. He did not encounter in a single case, he said, the microbe which Dr. Freire has described, and with which he professes to practice protective inoculations. He has, however, encountered this micrococcus in cultures made from the surface of the body, and believes its presence in Dr. Freire's blood cultures from the finger to have been quite accidental and without special significance.

Having proved by his microscopical researches and culture experiments that there is no specific germ in the blood of yellow fever patients, Dr. Sternberg turned his attention to the alimentary canal, and, as was to have been expected, encountered a variety of micro-organisms, some of which were apparently undescribed species, and therefore possible yellow fever germs. Among these is the bacillus of Dr. Paul Gibier, which was found in three out of ten cases. Dr. Sternberg is of opinion that Dr. Gibier has not as yet given any satisfactory proof that this is the veritable yellow fever germ, and further researches are required to determine the important questions relating to the cause and prevention of this disease. Dr. Sternberg has himself discovered several new micro-organisms, and it is possible that one or the other of these is the deadly microbe which he has so long been in search of, but he said he was not at present in a position to make a definite claim with reference to any one of them.

